# Personality and cancer survival: the Miyagi cohort study

**DOI:** 10.1038/sj.bjc.6602610

**Published:** 2005-05-17

**Authors:** N Nakaya, Y Tsubono, Y Nishino, T Hosokawa, S Fukudo, D Shibuya, N Akizuki, E Yoshikawa, M Kobayakawa, M Fujimori, K Saito-Nakaya, Y Uchitomi, I Tsuji

**Affiliations:** 1 Psycho-Oncology Division, National Cancer Center Research Institute East, 6-5-1 Kashiwanoha, Kashiwa, Japan; 2 Division of Epidemiology, Department of Public Health and Forensic Medicine, Tohoku University Graduate School of Medicine, 2-1 Seiryo, Sendai, Japan; 3 Department of Behavioral Medicine, Tohoku University Graduate School of Medicine, 2-1 Seiryo, Sendai, Japan; 4 Department of Human Development Disability, Tohoku University Graduate School of Education, 27-1 Kawauchi, Sendai, Japan; 5 Miyagi Cancer Society, Kamisugi 5-7-30, Sendai, Japan

**Keywords:** death from all causes, Eysenck personality questionnaire-revised, Japanese, prospective cohort study, survival

## Abstract

We tested the hypothesis that personality plays a role in cancer outcome in a population-based prospective cohort study in Japan. In July 1990, 41 442 residents of Japan completed a short form of the Eysenck Personality Questionnaire-Revised and a questionnaire on various health habits, and between January 1993 and December 1997, 890 incident cases of cancer were identified among them. These 890 cases were followed up until March 2001, and a total of 356 deaths from all causes was identified among them. Cox proportional-hazards regression was used to estimate the hazard ratio (HR) of death according to four score levels on each of four personality subscales (extraversion, neuroticism, psychoticism, and lie), with adjustment for potential confounding factors. Multivariable HRs of deaths from all causes for individuals in the highest score level on each personality subscale compared with those at the lowest level were 1.0 for extraversion (95% CI=0.8–1.4; Trend *P*=0.73), 1.1 for neuroticism (0.8–1.6; Trend *P*=0.24), 1.2 for psychoticism (0.9–1.6; Trend *P*=0.29), and 1.0 for lie (0.7–1.5; Trend *P*=0.90). The data obtained in this population-based prospective cohort study in Japan do not support the hypothesis that personality is associated with cancer survival.

Psychological factors may alter immune and endocrine function with possible effects on cancer incidence and outcome (Kiecolt-Glaser and Glaser, 1999; Spiegel and Kato, 1996), and the possibility that personality plays a role in cancer aetiology and outcome has often been raised (Garssen and Goodkin, 1999). The results of our earlier study, however, do not support the hypothesis that personality traits measured by the Eysenck questionnaire (extroversion, neuroticism, psychoticism, and lie) are risk factors for cancer (Nakaya *et al*, 2003).

The role of personality in the causation of cancer survival has been controversial. Of the four studies of cancer survival using the Eysenck questionnaire, one found statistically significant associations between higher scores for lie and increased risk of death in Hodgkin's disease or non-Hodgkin's lymphoma patients (Ratcliffe *et al*, 1995) and lower scores for extraversion (Hislop *et al*, 1987) (Ratcliffe *et al*, 1995) and breast cancer patients (Hislop *et al*, 1987). Two others found no association between extraversion or neuroticism and death in postoperative breast cancer patients (Greer *et al*, 1979; Dean and Surtees, 1989). However, these studies had several limitations, including failure to control for cigarette smoking or alcohol consumption, and failing to assess personality before the cancer diagnosis.

We conducted a population-based prospective cohort study in Japan to further investigate associations between the personality traits and cancer survival. To our knowledge, this is the first such study to assess personality before the diagnosis of cancer.

## MATERIALS AND METHODS

### Study cohort

We have reported the design of this prospective cohort study in detail elsewhere (Fukao *et al*, 1995; Nakaya *et al*, 2003; Suzuki *et al*, 2004). Briefly, we delivered two self-administered questionnaires to all 51 921 residents aged 40–64 years in 14 municipalities of Miyagi Prefecture in rural northern Japan from June through August 1990. The first questionnaire asked about various health habits, and the second was the Japanese version of the Eysenck Personality Questionnaire-Revised (EPQ-R) Short Form (Hosokawa and Ohyama, 1993). The questionnaires were delivered to, and collected at, the subjects’ residences by members of health promotion committees appointed by the municipal governments. The response rate for the first questionnaire was 91.7% (*n*=47 605), and that for the second questionnaire among the respondents to the first was 79.8% (*n*=41 442). The study protocol was approved by the institutional review board of Tohoku University Graduate School of Medicine. We considered the return of self-administered questionnaires signed by the subjects to imply their consent to participate in the study.

### Exposure data

The first questionnaire asked about demographic variables, personal and family histories of cancer and other diseases, and health habits, including smoking, alcohol use, diet, use of health services, and self-reported height and weight. The second questionnaire was a Japanese translation of the original English version of the EPQ-R Short Form, one of the series of personality inventories developed by Eysenck and Eysenck (1975). It contains 48 questions with dichotomised responses (yes or no); there are 12 questions for each of the four subscales (extraversion, neuroticism, psychoticism, and lie). Scores on each subscale ranged from 0 to 12, with higher scores indicated a greater tendency to possess the personality trait represented by each subscale. Extraversion represents sociability, liveliness, and surgency; neuroticism represents emotional instability and anxiousness; Psychoticism represents tough-mindedness, aggressiveness, coldness, and egocentricity; and lie represents unsophisticated dissimulation and social naivety or conformity (Eysenck and Eysenck, 1991).

In a previous study, we developed the Japanese version of the questionnaire and evaluated its reproducibility and validity among 329 college students and 253 adults (Hosokawa and Ohyama, 1993). Cronbach's alpha coefficient, a measure of internal consistency, was greater than 0.70 for all subscales except psychoticism (0.42 for college students and 0.48 for adults). The test–retest reliability coefficients of the four subscales over a 6-month period ranged from 0.70 to 0.85, indicating substantial stability. Confirmatory factor analysis supported the original theoretical structure of the four subscales proposed by Eysenck and Eysenck (1975). Scores on the four subscales were highly correlated with scores on similar subscales in the Japanese versions of the Sixteen Personality Factor Questionnaire (Izawa *et al*, 1982) and the Maudsley Personality Inventory (MPI Kenkyukai, 1969), indicating that the questionnaire had a high degree of concurrent validity.

### Follow-up

Population registries in the 14 municipalities were used to ascertain the vital and residential status of the subjects from 1 June 1990 through 31 December 1997, and 1609 incident cases of cancer were identified by computerised record linkage with the Miyagi Prefectural Cancer Registry covering the study area (Takano and Okuno, 1997). We excluded subjects with cancer diagnosed in the first 3 years from the baseline (*n*=451) because clinical stage of cancer was not recorded in the Miyagi Prefectural Cancer Registry for cases registered between June 1990 and December 1992.

Among the 1158 incident cases of cancer, we excluded subjects who responded ‘yes’ or ‘no’ to all 48 items on the EPQ-R, or failed to respond to any of them (*n*=168). We also excluded 100 subjects who indicated that they had completed the two questionnaires with the assistance of family members, because we suspected that might have affected the response patterns. Ultimately, data for 890 subjects (505 men and 385 women), with 356 deaths from all causes (241 men and 115 women), were used in the analysis.

### Statistical analysis

We counted person-years of follow-up for each subject from the date of diagnosis of cancer (from 1 January 1993 through 31 December 1997) until the date of emigration from the study districts, or the end of the study period (31 March 2001), whichever occurred first. A total of 3331 person-years accrued, and mean (s.d.) was 3.7 (2.5) years per person. Owing to logistical limitations, we discontinued follow-up of subjects who emigrated from the study municipalities, and as a result 22 subjects (2.5% of the analytic cohort) were lost to follow-up during the study period.

The numbers of subjects differed for each personality subscale, because subjects were excluded from the analyses of any subscale if answers to any of the questions on that subscale were missing. The number of subjects whose data were used for the analysis of extraversion, neuroticism, psychoticism, and lie was 825, 822, 819, and 819, respectively. The scores on each personality subscale (scored on a scale of 0–12) were divided into four score levels approximately equal in size based on the scores of the subjects as a whole. As a result, the cutoff scores vary with the subscale. Hazard ratios (HRs) were computed as the death rate among subjects in each score level of a personality subscale divided by the death rate among subjects in the lowest score level. Hazard ratios were also estimated by analysing deaths from all causes and deaths from cancer.

We used the SAS PHREG procedure on SAS version 8.2 statistical software package (Cary, NC, USA) to evaluate Cox proportional-hazard regression to adjust for sex, age in years at cancer diagnosis, and other potentially confounding variables (Rothman and Greenland, 1998). *P*-values for testing statistical significance of linear trends were calculated by treating personality subscales as continuous variables. All *P*-values were two-tailed.

In addition to sex and age in years at cancer diagnosis, we considered the following variables to be potential confounders *a priori:* clinical stage (*in situ* or localised, lymph node metastasis, regional invasion, distant metastasis); surgery (with excision of the primary lesion or without excision of the primary lesion); method of diagnosis (mass screening and health checkup or other); initial cancer site (stomach, colorectum, lung, breast, or other sites); past history (stroke, myocardial infarction, hypertension, diabetes mellitus); education (up to 15 years of age, from 16 to 18 years, 19 years or older); marital status (whether or not living with spouse); cigarette smoking (never smoker, past smoker, current smoker); alcohol consumption (never drinker, past drinker, current drinker). This study used the clinical staging system developed by the Research Group for Population-Based Cancer Registration in Japan. Lesions at each cancer site were classified into four stages (*in situ* or localised, lymph node metastasis, regional invasion, distant metastasis) based on the SEER Program (Surveillance, Epidemiology, and End Results Program) and the UICC-TNM classification (Research Group for Population-Based Cancer Registration in Japan, 1999). We further assessed associations between clinical stage and risk of death from all causes.

Stratified analyses were performed according to sex, age group in years at cancer diagnosis (10-year age class), clinical stage, surgery, occasion of diagnosis, past history (stroke, myocardial infarction, hypertension, or diabetes mellitus), cigarette smoking, alcohol consumption, education, marital status, and initial major cancer sites. The four most common cancer sites among the subjects, namely, stomach, colorectum, lung, and breast, were used as the major cancer sites. Combinations of cancer sites consisting of digestive organs or respiratory organs were also used (World Health Organization, 1992; Schapiro *et al*, 2001).

## RESULTS

We first compared the characteristics of the subjects in the highest and lowest levels (i.e., approximate quartiles) of score on each personality subscale (Table 1). Subjects in the highest level for extraversion were more likely to live with a spouse and current smoker and less likely to have a past history of hypertension; those in the highest level for neuroticism were more likely to be female, have *in situ* or localised as their clinical stage, and a past history of hypertension, and less likely to be living with a spouse and be a current smoker. Subjects in the highest level for psychoticism were more likely to be a man, current smoker, and current drinker. Subjects in the highest level for lie were older, more likely to be a woman, to have been diagnosed by mass screening or a health checkup, and less likely to be living with a spouse, be a current smoker, or current drinker.

The clinical stages of the cancers were strongly associated with risk of death from all causes: The multivariate HRs for *in situ* or localised (95% CI) were 3.2 (1.9–5.3), 5.4 (3.4–8.4), and 7.3 (4.8–11.3) in subjects with lymph node metastasis, regional invasion, and distant metastasis, respectively.

The multivariable Cox proportional-hazards regression analyses showed no significant associations between any of the four personality subscales and the HR of death from all causes (Table 2), and these findings remained basically unchanged when we tested for associations between four personality subscales and the risk of death from cancer. We divided each personality subscale into tertiles or quintiles as closely as possible so our observations were quite similar and replicated (data not shown).

Stratified analyses according to sex, age group in years at cancer diagnosis, clinical stage, surgery, occasion of diagnosis, past history, education, marital status, cigarette smoking, and alcohol consumption showed that the associations between personality subscales and risk of death from all causes were not markedly affected by any of these variables (data not shown).

The multivariable Cox proportional-hazards regression analyses also showed no significant associations between personality subscales and risk of death from all causes stratified by the four major cancer sites (stomach, colorectum, lung, and breast) (Table 3). For stomach cancer, extraversion and neuroticism showed slightly (but not significantly) higher risk of death from all causes. For lung cancer, neuroticism showed a slightly lower (nonsignificant) risk of death from all causes. No significant associations were also found in the combinations of cancer sites (digestive and respiratory organs).

## DISCUSSION

This population-based prospective cohort study in rural Japan revealed no significant association between any of the four personality traits measured by the Eysenck questionnaire (extraversion, neuroticism, psychoticism, and lie) in cancer patients and risk of death from all causes.

Four earlier studies investigated association between scores on the subscale of the Eysenck questionnaire and cancer survival. One followed up 63 cases of Hodgkin's disease or non-Hodgkin's lymphoma for 5 years, with 27 deaths from all causes and found a significant positive, linear association between lie and the risk of death from all causes (trend *P*=0.001) (Ratcliffe *et al*, 1995). Another followed up 121 postoperative cases of breast cancer for 6–8 years, identifying 22 deaths from all causes but found no significant associations with extraversion, neuroticism, or lie (Dean and Surtees, 1989). In a follow up of 133 cases of breast cancer for 4 years, with 26 deaths from all causes, a significant negative association was observed between extraversion and the risk of death (multivariate HR=0.33, *P*<0.05) (Hislop *et al*, 1987). Neuroticism was not associated with risk of death (multivariate HR=0.85, *P*>0.05). Lastly, in a follow up of 69 cases of breast cancer for 5 years with 18 deaths from all causes, there were no significant associations with extraversion or neuroticism (Greer *et al*, 1979).

Other studies have mainly focused on the possible association between psychological factors, including component factors of type C personality (Temoshok *et al*, 1985), such as emotional suppression, helplessness/hopelessness, and cancer survival. In a recent large prospective cohort study, 578 cases of early-stage breast cancer were followed up for at least 5 years with 133 deaths from all causes; emotional suppression and helplessness/hopelessness did not increase the risk of death (Watson *et al*, 1999). There is also little consistent evidence from other studies that these factors are important in cancer survival (Petticrew *et al*, 2002). Our findings also suggested that Eysenck personality dimensions do not substantially influence cancer survival.

Our study had several methodologic advantages over previous studies of personality and cancer survival. First, we controlled extensively for potential confounding variables, including cigarette smoking and alcohol consumption. Various studies have reported that smoking or alcohol consumption are associated with extraversion or other personality characteristics (McManus and Weeks, 1982; Arai *et al*, 1997; Vollrath and Torgersen, 2002; Nakaya *et al*, 2003) as well as cancer outcome (Deleyiannis *et al*, 1996; Oh *et al*, 2000; Tammemagi *et al*, 2004). We observed little differences in the point estimates of the HRs according to whether multivariate adjustments were made for smoking and other variables (Table 2). Second, we assessed the responses to the personality questionnaire before the diagnosis of cancer, and to our knowledge this is the first study on associations between personality before cancer diagnosis and the risk of death from all causes. All earlier studies assessed the responses to personality questionnaire after the cancer diagnosis, and the personality scores may have been influenced by the cancer diagnosis or by treatment. Our assessment of personality was free from such distortions.

One strength of our study is the exclusion of subjects with cancer in the first 3 years of follow-up, because clinical stage was not recorded in the Miyagi Prefectural Cancer Registry for cases registered between June 1990 and December 1992. This would have improved the validity of our findings, since such cancers diagnosed soon after the baseline personality assessment might have distorted responses because of to subclinical symptoms caused by as-yet diagnosed cancers.

Limitations of our study include the small number of subjects with cancer at specific sites. Although we found no significant association between any of the four personality trait and risk of death among the subjects with the commonest cancers, the analyses may not have had sufficient statistical power to detect associations between small increases or decreases in the risk of death from all causes at individual sites and personality traits. Third, we had no information on health behaviours after the cancer diagnosis or on compliance with treatment.

In conclusion, the data obtained in this population-based prospective cohort study in rural Japan do not support the hypothesis that personality is associated with cancer survival. We suggest that cancer patients should not feel pressured into adopting a particular personality to improve their survival.

## Figures and Tables

**Table 1:** Characteristics of the subjects according to scores in the highest and lowest of four score levels of each of personality subscales

	**Personality subscale score levels^a^**							
	**Extraversion (*n*=825)**		**Neuroticism (*n*=822)**		**Psychoticism (*n*=819)**		**Lie (*n*=819)**	
**Characteristics**	**⩽3**	**⩾9**	**⩽3**	**⩾9**	**⩽3**	**⩾6**	**⩽5**	**⩾10**
No.	248	172	243	174	277	205	181	202
Men (%)	59.7	59.3	60.5	55.2	39.4	79.2	69.6	47.0
*Cancer registration at cancer diagnosis*								
Age in years at cancer diagnosis, means±s.d.	60.6±6.9	60.9±6.5	61.3±7.0	60.0±7.5	60.5±7.2	60.2±6.8	58.6±7.6	62.5±5.7
Initial cancer site (%)								
Stomach	22.2	23.3	22.2	23.0	19.9	25.9	22.1	20.3
Colorectum	18.6	16.9	18.1	16.1	15.5	16.1	17.1	18.3
Lung	13.3	10.5	14.4	10.3	9.8	12.2	9.9	14.9
Breast	10.1	10.5	7.8	12.1	12.6	6.3	9.4	7.9
Other sites	35.9	39.0	37.5	38.5	42.2	39.5	41.4	38.6

Clinical stage (%)								
*In situ* or localised	37.7	34.9	35.4	44.8	40.4	34.2	40.9	35.1
Lymph node metastasis	8.5	11.6	12.4	8.0	10.1	12.7	7.7	10.9
Regional invasion	8.9	7.6	9.5	8.6	9.4	6.8	10.5	7.9
Distant metastasis	12.6	12.2	11.9	10.3	10.8	12.7	11.1	11.9
Unknown	32.4	33.7	30.9	28.2	29.2	33.7	29.8	34.2

Surgery (%)								
Surgery with excision of the primary lesion	66.9	70.4	68.3	69.0	71.5	64.4	66.3	68.8
Surgery without excision of the primary lesion	17.3	16.3	16.0	17.2	15.9	19.0	18.8	15.8
Unknown	15.7	13.4	15.6	13.8	12.6	16.6	14.9	15.3

Occasion of diagnosis (%)								
Mass screening or health checkup	28.2	27.3	30.5	28.2	29.6	23.9	24.9	31.7
Other	48.8	50.0	48.6	46.0	47.7	52.7	53.0	45.0
Unknown	23.0	22.7	21.0	25.9	22.7	23.4	22.1	23.3

*Self-report questionnaire at 1990*								
Past history (%)								
Stroke	0.4	0.0	0.4	0.6	0.0	1.5	1.1	0.0
Myocardial infarction	0.0	1.7	2.1	1.2	1.1	2.4	2.8	2.0
Hypertension	30.2	20.9	18.9	28.2	28.2	26.3	26.0	22.8
Diabetes mellitus	6.1	5.2	4.1	6.9	5.8	7.3	7.2	5.5
Education, in school until age 19 years or older (%)	13.7	15.1	12.4	12.1	15.9	7.8	11.1	12.4
Marital status, living with spouse (%)	79.8	85.5	88.1	79.3	83.8	83.9	89.0	82.2
Current smoker (%)	38.3	44.2	47.3	39.1	27.4	59.5	52.5	31.7
Current drinker (%)	51.2	55.8	56.4	55.2	43.0	63.4	65.8	38.1

a The scores on each personality subscale (scored on a scale of 0–12) were divided into four score levels approximately equal in size based on the scores of the subjects as a whole, and as a result the cutoff scores vary with the subscale.

**Table 2:** Results of Cox proportional-hazards regression analysis of EPQ-R subscales scores and cancer survival^a^

	**Personality subscale score levels** b				
	**1 (reference)**	**2**	**3**	**4**	**Trend *P*-value^c^**
*Extraversion (n*=*825)*					
Score	⩽3	4–5	6–8	⩾9	
Person-years of follow-up	943	704	844	659	
No. of death (all-cause/cancer)	97/88	63/60	93/85	69/63	
Age-, sex-adjsuted HR1	1.0	0.9 (0.7–1.3)	1.1 (0.8–1.5)	1.0 (0.7–1.4)	0.62
Multivariable HR1	1.0	0.8 (0.6–1.1)	1.1 (0.8–1.4)	1.1 (0.8–1.4)	0.73
Multivariable HR2	1.0	0.8 (0.6–1.1)	1.1 (0.8–1.5)	1.0 (0.7–1.5)	0.65

*Neuroticism (n*=*822)*					
Score	⩽3	4–5	6–8	⩾9	
Person-years of follow-up	899	700	835	674	
No. of death (all-cause/cancer)	96/86	58/54	104/99	68/62	
Age-, sex-adjsuted HR1	1.0	0.8 (0.6–1.2)	1.1 (0.9–1.5)	1.0 (0.8–1.4)	0.32
Multivariable HR1	1.0	0.9 (0.6–1.3)	1.1 (0.8–1.4)	1.1 (0.8–1.6)	0.24
Multivariable HR2	1.0	0.9 (0.7–1.3)	1.1 (0.8–1.5)	1.2 (0.8–1.7)	0.17

*Psychoticism (n*=*819)*					
Score	⩽3	4	5	⩾6	
Person-years of follow-up	1100	787	482	700	
No. of death (all-cause/cancer)	97/89	81/78	50/47	97/90	
Age-, sex-adjsuted HR1	1.0	1.1 (0.8–1.5)	1.0 (0.7–1.5)	1.2 (0.8–1.7)	0.20
Multivariable HR1	1.0	1.1 (0.8–1.5)	0.9 (0.7–1.3)	1.2 (0.9–1.6)	0.29
Multivariable HR2	1.0	1.2 (0.8–1.6)	1.0 (0.7–1.4)	1.2 (0.9–1.7)	0.31

*Lie (n*=*819)*					
Score	⩽5	6–7	8–9	⩾10	
Person-years of follow-up	660	745	923	754	
No. of death (all-cause/cancer)	72/66	68/64	105/99	79/71	
Age-, sex-adjsuted HR1	1.0	0.9 (0.6–1.3)	1.0 (0.8–1.4)	0.9 (0.7–1.3)	0.61
Multivariable HR1	1.0	0.9 (0.6–1.2)	1.0 (0.7–1.4)	1.0 (0.7–1.5)	0.90
Multivariable HR2	1.0	0.9 (0.6–1.3)	1.0 (0.7–1.4)	1.0 (0.7–1.5)	0.88

a The multivariate hazard ratio (HR) has been adjusted for sex; age in years at cancer diagnosis (40–49, 50–59, or 60 years or older); clinical stage *(in situ* or localised, lymph node metastasis, regional invasion, distant metastasis); surgery (with excision of the primary lesion or without excision of the primary lesion); occasion of diagnosis (mass screening and health checkup, others); initial cancer site (stomach, colorectum, lung, breast, other sites); past history (stroke, myocardial infarction, hypertension, diabetes mellitus); education (up to 15 years of age, from 16 to 18 years, 19 years or older); marital status (whether living with a spouse); cigarette smoking (never smoker, past smoker, or current smoker); alcohol consumption (never drinker, past drinker, or current drinker). HR1=hazard ratio of death from all causes as an end point; HR2=hazard ratio of death from cancer as an end point. All HRs are reported with the 95% confidence interval (CI) in parentheses. EPQ-R=Eysenck Personality Questionnaire-Revised (EPQ-R) Short Form.

b The scores on each personality subscale (scored on a scale of 0–12) were divided into four score levels approximately equal in size based on the scores of the subjects as a whole. As a result, the cutoff scores vary with the subscale.

c *P*-values for linear trend were calculated by treating personality subscales as continuous variables.

**Table 3:** Multivariable hazard ratios (HRs) of major cancer sites or combinations of cancer sites in the highest of the four score levels as compared with the lowest level of each of four personality subsacales^a^

	**Extraversion**		**Neuroticism**		**Psychoticism**		**Lie**	
	**HR**	**Trend *P*-value^b^**	**HR**	**Trend *P*-value^b^**	**HR**	**Trend *P*-value^b^**	**HR**	**Trend *P*-value^b^**
*Major cancer sites (No. of subjects/deaths from all-cause)* c								
Stomach	182/59		179/60		180/61		178/59	
Multivariable HR	1.5 (0.7–3.5)	0.32	1.8 (0.9–3.9)	0.15	0.9 (0.4–1.7)	0.56	1.3 (0.7–2.4)	0.37

Colorectum	153/39		153/40		153/40		150/42	
Multivariable HR	0.5 (0.2–1.3)	0.17	1.0 (0.3–3.0)	0.36	0.9 (0.2–3.0)	0.78	0.7 (0.3–1.3)	0.32

Lung	91/59		95/63		87/59		89/58	
Multivariable HR	0.9 (0.3–2.4)	0.65	0.4 (0.1–1.1)	0.94	1.1 (0.4–3.1)	0.77	1.2 (0.4–3.1)	0.77

Breast	77/11		73/10		80/12		74/11	
Multivariable HR	0.5 (0.0–31.2)	0.20	Not applicable		Not applicable		Not applicable	

*Combinations of cancer sites (No. of subjects/deaths from all-cause)* c								
Digestive organs^d^	370/118		364/120		366/121		361/121	
Multivariable HR	0.9 (0.5–1.6)	0.90	0.9 (0.5–1.4)	0.88	0.8 (0.5–1.4)	0.32	1.0 (0.5–1.8)	0.59

Respiratory organs^e^	108/61		113/65		105/61		107/60	
Multivariable HR	0.9 (0.3–3.0)	0.96	0.5 (0.2–1.6)	0.78	1.2 (0.5–2.2)	0.07	0.8 (0.3–2.4)	0.80

a The multivariate hazard ratio (HR) has been adjusted for sex; age in years at cancer diagnosis (40–49, 50–59, or 60 years or older); clinical stage (*in situ* or localised, lymph node metastasis, regional invasion, distant metastasis); surgery (with excision of the primary lesion or without excision of the primary lesion); occasion of diagnosis (mass screening and health checkup, others); past history (stroke, myocardial infarction, hypertension, diabetes mellitus); education (up to 15 years of age, from 16 to 18 years, 19 years or older); marital status (whether living with a spouse); cigarette smoking (never smoker, past smoker, or current smoker); alcohol consumption (never drinker, past drinker, or current drinker). Hazard ratio denotes hazard ratio of death from all causes as an end point. All HRs are reported with the 95% confidence interval (CI) in parentheses.

b *P*-values for linear trend were calculated by treating personality subscales as continuous variables.

c The total numbers of subjects or deaths from all causes were used in the analyses.

d Digestive organs: oesophagus, stomach, small intestine, colon, rectum, and anus.

e Respiratory organs: nasal cavity, middle ear, accessory sinuses, larynx, trachea, bronchus, and lung.

## References

[bib1] Arai Y, Hosokawa T, Fukao A, Izumi Y, Hisamichi S (1997) Smoking behaviour and personality: a population-based study in Japan. Addiction 92: 1023–1033 9376772

[bib2] Dean C, Surtees G (1989) Do psychological factors predict survival in breast cancer? J Psychosom Res 33: 561–569 2795528 10.1016/0022-3999(89)90063-9

[bib3] Deleyiannis FW, Thomas DB, Vaughan TL, Davis S (1996) Alcoholism: independent predictor of survival in patients with head and neck cancer. J Natl Cancer Inst 88: 542–549 8606383 10.1093/jnci/88.8.542

[bib4] Eysenck HJ, Eysenck SBG (1975) Manual of the Eysenck Personality Questionnaire (Adult and Junior). London (UK): Hodder & Stoughton

[bib5] Eysenck HJ, Eysenck SBJ (1991) Manual of the Eysenck Personality Scales. (EPS Adult). London (UK): Hodder & Stoughton

[bib6] Fukao A, Tubono Y, Komatsu S, Tsuji I, Minami Y, Hisamichi S, Hosokawa T, Kawamura M, Takano A, Sugihara N, Ikeda T, Nisikori M (1995) Cohort study on the relation of lifestyle, personality and biologic markers to cancer in Miyagi, Japan: study design, response rate and profiles of the cohort subjects. J Epidemiol 5: 153–157

[bib7] Garssen B, Goodkin K (1999) On the role of immunological factors as mediators between psychosocial factors and cancer progression. Psychiat Res 85: 51–61 10.1016/s0165-1781(99)00008-6 10195316

[bib8] Greer S, Morris T, Pettingale KW (1979) Psychosocial response to breast cancer: effect on outcome. Lancet i: 931–932 10.1016/s0140-6736(79)92127-5 90871

[bib9] Hislop TG, Waxler NE, Coldman AJ, Elwood JM, Kan L (1987) The prognostic significance of psychosocial factors in women with breast cancer. J Clin Epidemiol 40: 729–735 10.1016/0021-9681(87)90110-x 3597675

[bib10] Hosokawa T, Ohyama M (1993) Reliability and validity of the Japanese version of the short form Eysenck Personality Questionnaire-Revised. Psychol Rep 72: 823–832

[bib11] Izawa S, Yamaguchi K, Tatsuoka M, Motigi M, Uchiyama T, Ueno K (1982) Manual of the Sixteen Personality Factor Questionnaire (Japanese version). Tokyo (Japan): Nihon Bunka Kagakusha (In Japanese)

[bib12] Kiecolt-Glaser JK, Glaser R (1999) Psychoneuroimmunology and cancer: fact or fiction? Eur J Cancer 35: 1603–1607 10673969 10.1016/s0959-8049(99)00197-5

[bib13] McManus IC, Weeks SJ (1982) Smoking, personality and reasons for smoking. Psychol Med 12: 349–356 7100358 10.1017/s0033291700046687

[bib14] MPI Kenkyukai (1969) Shin Seikaku Kensahou [Manual of the Maudsley Personality Inventory, Japanese version]. Tokyo (Japan): Seishin Shobo (In Japanese)

[bib15] Nakaya N, Tsubono Y, Hosokawa T, Nishino Y, Ohkubo T, Hozawa A, Shibuya D, Fukudo S, Fykao A, Tsuji I, Hisamichi S (2003) Personality and the risk of cancer. J Natl Cancer Inst 95: 799–805 12783934 10.1093/jnci/95.11.799

[bib16] Oh WK, Manola J, Renshaw AA, Brodkin D, Loughlin KR, Richie JP, Shapiro CL, Kantoff PW (2000) Smoking and alcohol use may be risk factors for poor outcome in patients with clear cell renal carcinoma. Urology 55: 31–35 10.1016/s0090-4295(99)00408-2 10654890

[bib17] Petticrew M, Bell R, Hunter D (2002) Influence of psychological coping on survival and recurrence in people with cancer: systematic review. BMJ 325: 1066–1075 12424165 10.1136/bmj.325.7372.1066 PMC131179

[bib18] Ratcliffe MA, Dawson AA, Walker LG (1995) Eysenck Personality Inventory *L*-scores in patients with Hodgkin's disease and non-Hodgkin's lymphoma. Psychooncology 4: 39–45

[bib19] Research Group for Population-Based Cancer Registration in Japan (Chairman: Oshima A) (1999) Guidelines on Population-Based Cancer Registration in Japan Fourth Revision. Osaka: Suehiroinsatsu, pp 172–191, (In Japanese)

[bib20] Rothman KJ, Greenland S (1998) Modern Epidemiology 2nd edn. Philadelphia (PA): Lippincott-Raven, pp 359–399

[bib21] Schapiro IR, Ross-Petersen L, Saelan H, Garde K, Olsen JH, Johansen C (2001) Extraversion and neuroticism and the associated risk of cancer: a Danish cohort study. Am J Epidemiol 153: 757–763 11296147 10.1093/aje/153.8.757

[bib22] Spiegel D, Kato PM (1996) Psychological influences on cancer incidence and progression. Harv Rev Psychiat 4: 10–26 10.3109/10673229609030518 9384968

[bib23] Suzuki Y, Tsubono Y, Nakaya N, Suzuki Y, Koizumi Y, Tsuji I (2004) Green tea and the risk of breast cancer: pooled analysis of two prospective studies in Japan. Br J Cancer 90: 1361–1363 15054454 10.1038/sj.bjc.6601652 PMC2409667

[bib24] Takano A, Okuno Y (1997) Japan, Miyagi prefecture. In Cancer Incidence in Five Continents Parkin DM, Whelan SL, Ferlay J, Raymond L, Young J (eds), Vol. 7. France: Lyon, IARC Sci Publ143: 386–389

[bib25] Tammemagi CM, Neslund-Dudas C, Simoff M, Kvale P (2004) Smoking and lung cancer survival: the role of comorbidity and treatment. Chest 125: 27–37 14718417 10.1378/chest.125.1.27

[bib26] Temoshok L, Heller BW, Sagebiel RW, Blois MS, Sweet DM, DiClemente RJ, Gold ML (1985) The relationship of psychosocial factors to prognostic indicators in cutaneous malignant melanoma. J Psychosom Res 29: 139–153 4009515 10.1016/0022-3999(85)90035-2

[bib27] Vollrath M, Torgersen S (2002) Who takes health risks? A probe into eight personality types. Pers Individ Diff 32: 1185–1197

[bib28] Watson M, Haviland JS, Greer S, Davidson J, Bliss JM (1999) Influence of psychological response on survival in breast cancer: a population-based cohort study. Lancet 354: 1331–1336 10533861 10.1016/s0140-6736(98)11392-2

[bib29] World Health Organization (1992) International Statistical Classification of Diseases and Related Health Problems. 10th Revision. Geneva: World Health Organization, pp 107–181

